# Pairwise Performance Comparison of Docking Scoring Functions: Computational Approach Using InterCriteria Analysis

**DOI:** 10.3390/molecules30132777

**Published:** 2025-06-27

**Authors:** Maria Angelova, Petko Alov, Ivanka Tsakovska, Dessislava Jereva, Iglika Lessigiarska, Krassimir Atanassov, Ilza Pajeva, Tania Pencheva

**Affiliations:** Institute of Biophysics and Biomedical Engineering, Bulgarian Academy of Sciences, 105 Acad. G. Bonchev Str., 1113 Sofia, Bulgaria; maria.angelova@biomed.bas.bg (M.A.); petko@biophys.bas.bg (P.A.); itsakovska@biomed.bas.bg (I.T.); dessislava.jereva@biomed.bas.bg (D.J.); iglika@biomed.bas.bg (I.L.); k.t.atanassov@gmail.com (K.A.); pajeva@biomed.bas.bg (I.P.)

**Keywords:** molecular operating environment, scoring functions, molecular docking, InterCriteria analysis

## Abstract

Scoring functions are key elements in docking protocols as they approximate the binding affinity of a ligand (usually a small bioactive molecule) by calculating its interaction energy with a biomacromolecule (usually a protein). In this study, we present a pairwise comparison of scoring functions applying a multi-criterion decision-making approach based on InterCriteria analysis (ICrA). As criteria, the five scoring functions implemented in MOE (Molecular Operating Environment) software were selected, and their performance on a set of protein–ligand complexes from the PDBbind database was compared. The following docking outputs were used: the best docking score, the lowest root mean square deviation (RMSD) between the predicted poses and the co-crystallized ligand, the RMSD between the best docking score pose and the co-crystallized ligand, and the docking score of the pose with the lowest RMSD to the co-crystallized ligand. The impact of ICrA thresholds on the relations between the scoring functions was investigated. A correlation analysis was also performed and juxtaposed with the ICrA. Our results reveal the lowest RMSD as the best-performing docking output and two scoring functions (Alpha HB and London dG) as having the highest comparability. The proposed approach can be applied to any other scoring functions and protein–ligand complexes of interest.

## 1. Introduction

The search for hit compounds with specific biological activity requires considerable effort and resources. The primary efforts concern the synthesis and biological testing of a large number of compounds. This defines the strong interest of the R&D pharmaceutical sector towards computer-aided methods that facilitate rational drug design [1]. Among these methods, molecular docking is particularly important when the structure of a biomacromolecule is known. This method is used to predict the binding affinity of a ligand in the active site (by employing scoring functions to calculate docking scores) based on the ligand docking poses (conformations and positions) in the binding site. Docking is also applied in virtual screening to identify potential active ligands for a specific macromolecular target among thousands to millions of compounds. As the calculations of docking scores can be time-consuming, the selection of appropriate scoring functions could impact the overall effectiveness of the computational procedure. Over the past decades, a number of studies have conducted comparative assessments of scoring functions implemented in both free and commercial docking software [2,3,4,5]. To evaluate the predictive power and examine the strengths and limitations of the scoring functions, several benchmark test sets have been developed [3,6,7]. These datasets are highly diverse, encompassing a wide range of protein families, ligand chemotypes, and binding affinities [8], thus providing good opportunities for the comparison of docking scoring functions. Despite the studies [2,3,4,5] assessing and comparing the scoring functions in docking, the question regarding which functions are more appropriate for use compared to others remains open.

In this study, the docking scoring functions implemented in Molecular Operating Environment (MOE, https://www.chemcomp.com/, accessed on 22 May 2025) [9], the widely used drug discovery software platform, were selected for comparative analysis. The MOE docking module operates with five scoring functions, namely London dG, ASE, Affinity dG, Alpha HB, and GBVI/WSA dG [8,9]. The first four are empirical, while GBVI/WSA dG is a force-field function [5]. Empirical functions evaluate the binding affinity of protein–ligand complexes based on a set of weights defined by multiple linear regression to experimentally measured affinities. The equation terms describe important contributions to the protein–ligand binding, like hydrogen bonding, ionic and hydrophobic interactions, loss in ligand flexibility, etc. On the other hand, the force-field-based scoring functions use classical force fields to evaluate protein–ligand interactions. In their simplest forms, Lennard-Jones and Coulomb potentials are applied to describe the enthalpy terms.

The comparison of MOE scoring functions in this research is based on the Comparative Assessment of Scoring Functions benchmark subset (CASF-2013) [3] of the PDBbind database [6], which is a comprehensive collection of 195 protein–ligand complexes with available binding affinity data. The CASF-2013 dataset was used for the assessment of scoring functions implemented in free docking software such as AutoDock and Vina [10]. In [11], the CASF-2013 dataset supported the evaluation of 12 scoring functions based on machine learning techniques. The high-quality set of 195 test complexes enabled the comparison and assessment of scoring functions embedded in leading software platforms like Schroedinger, MOE, Discovery Studio, SYBYL, and GOLD [12]. CASF-2013, along with another independent benchmark dataset named Community Structure-Activity Resource (CSAR 2014), was used in an evaluation analysis of an improved version of the scoring function SPecificity and Affinity (SPA) [7]. CASF-2013 was applied to validate 3D convolutional neural network (3D-CNN) scoring functions and end-to-end 3D-CNNs with a mechanism for spatial attention [13,14], as well as for extensive validation of the scoring power of geometric graph learning with extended atom-type features for protein–ligand binding affinity prediction [15]. Thus, CASF-2013 was selected as the dataset in our study.

The InterCriteria analysis (ICrA) [16] was applied for pairwise performance comparison of the MOE scoring functions on CASF-2013. The expectations were for the ICrA to reveal relations between the scoring functions by analyzing a variety of data outputs extracted from molecular docking. ICrA was elaborated as a multi-criterion decision-making approach that detects possible relations between pairs of criteria when multiple objects are considered. Over the past years of research, the approach has already demonstrated its potential when applied to economic, biomedical, industrial, and various other data sets and problem formulations [17]. One of the research objectives was to specify the general framework of the problems that may be solved by the approach, as well as the connections between the new approach and classical multi-criteria decision-making methods such as correlation analyses [https://intercriteria.net, accessed on 22 May 2025]. Some case studies of the ICrA applications are freely accessible [https://intercriteria.net/studies/, accessed on 22 May 2025], e.g., in relation to the World Economic Forum’s global competitiveness reports, genetic algorithm performance, ant colony optimization performance, and analysis of calorimetric data of blood serum proteome.

Recently, the ICrA approach was applied in the field of computer-aided drug design and computational toxicology in comparative studies of various scoring functions [18,19], as well as in in silico studies of biologically active molecules [20]. In contrast to our previous studies, here we apply ICrA on the CASF-2013 benchmark subset of the PDBbind database covering several important docking outputs. The current investigation is a significant extension of our previous studies [18,19,20] as it is based on a much larger dataset of structurally and functionally different proteins and their ligands, with a detailed investigation of the impact of key ICrA thresholds on the comparison results. A correlation analysis was also performed and juxtaposed with the ICrA results. Our results outline the most and the least similar scoring functions and the most comparable docking outputs. In addition, they demonstrate the applicability of ICrA to reveal new relations between the studied criteria.

## 2. Results and Discussion

For the CASF-2013 dataset, re-docking of the ligands in the protein–ligand complexes was performed. The following data were extracted from 30 saved poses:

(i) the best docking score (a lower score suggests better protein–ligand binding)—hereinafter referred to as BestDS;
(ii) the lowest root mean square deviation (RMSD) between the predicted poses and the ligand in the co-crystallized complex—hereinafter referred to as BestRMSD;
(iii) the RMSD between the pose with the best docking score and the ligand in the co-crystallized complex—hereinafter referred to as RMSD_BestDS;
(iv) the docking score of the pose with the lowest RMSD to the ligand in the co-crystallized complex—hereinafter referred to as DS_BestRMSD.

For the sake of completeness, we have also investigated the possible relations to the experimental data available in CASF-2013. Based on the availability, *K_d_* or *K_i_*, expressed as (−log*K_d_*) or (−log*K_i_*), respectively, were used.

The collected information was formatted for subsequent application of the ICrA: the ligands from protein–ligand complexes were considered objects of research in terms of the ICrA, while the different scoring functions (represented by BestDS, BestRMSD, RMSD_BestDS, or DS_BestRMSD), along with the binding affinity data, were considered as criteria.

ICrA was applied in two steps. Initially, it was implemented on the data outputs extracted from molecular docking results as described above, with the conditionally defined (default in ICrAData) values *α* = 0.75 and *β* = 0.25, based on which the ranges of *consonance* and *dissonance* are defined [21].

Table 1 summarizes the results obtained for the degrees of agreement *µ* between the five scoring functions of MOE and the binding affinity data of the studied complexes after the ICrA application. The colors in Table 1 reproduce the ones used in ICrAData (green represents *positive consonance*, magenta represents *dissonance*) for the conditionally defined values of *α* = 0.75 and *β* = 0.25. The ICrA relations between the scoring functions and experimental data are highlighted in grey at the bottom of the table.

As seen from Table 1, “varicolored” ICrA results were obtained only when BestRMSD was considered. For the rest of the docking outputs, ICrA does not outline any significant relations (other than in *dissonance*) between the five investigated scoring functions in MOE, as well as between the scoring functions and the binding affinity data—all hit the *dissonance* zone only (colored in magenta).

In the second step, the impact of the variations in *α* and *β* values on the investigated relations was explored. The thresholds α and *β* might be determined algorithmically or chosen by the user. Since these parameters define the thresholds for *positive consonance*/*dissonance*/*negative consonance* between the studied pairs of criteria, we investigated the impact of their values on the relations between the scoring functions. We varied the difference between *α* and *β* (keeping their sum at 1.00) starting from 0.5 (*α* = 0.75 and *β* = 0.25) and decreasing it gradually to 0.2 (*α* = 0.6 and *β* = 0.4) to follow how these values affect the relations between the investigated scoring functions (for clarity, these analyses are reported using ICrA colors in Supplementary Tables S1–S5). The results are summarized in Table 2 and Table 3 based on the following two indicators: the number of pairs of scoring functions in *positive consonance* for each docking output (Table 2) and the number of docking outputs in *positive consonance* for each pair of scoring functions (Table 3).

The first indicator, the number of pairs of scoring functions in *positive consonance*, could be considered as a measure of the “sensitivity” of the corresponding docking output to the *α* and *β* values. The second one, the number of docking outputs for each pair in *positive consonance*, allows for a comparison of the scoring functions based on the number of the docking outputs—a higher number of docking outputs in *positive consonance* indicates a more similar performance of the pairs of scoring functions studied.

As expected, the decrease in the difference between *α* and *β* results in a higher number of pairs in *positive consonance*, thus raising the question regarding the most appropriate *α* and *β* values. The values of 0.67/0.33 and 0.65/0.35 show almost identical results, and 0.67/0.33 appears to be a good compromise between “no” and “many” pairs in *positive consonance*, also allowing for a certain tolerance in comparability between the studied criteria.

**Figure 1:**
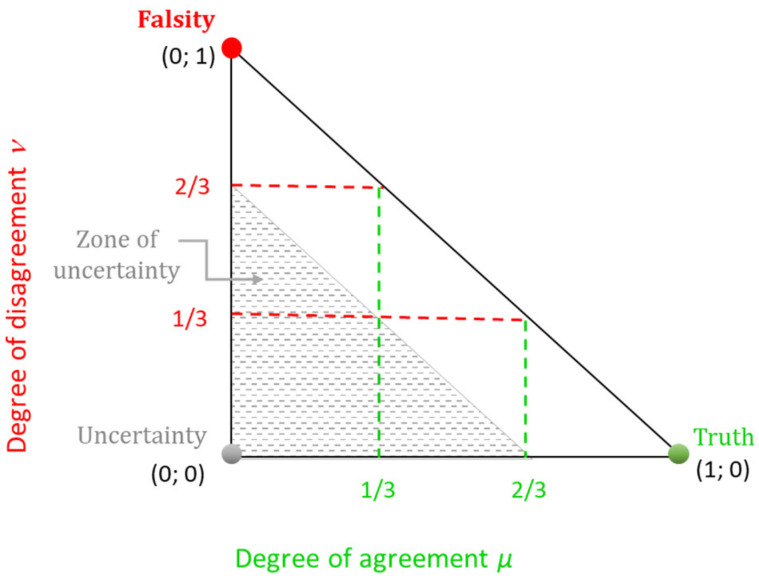
Interpretative intuitionistic triangle in the case of “thirds”.

In addition, the values *α* = 0.67 and *β* = 0.33 allow for the presentation of an alternate scale for *consonance*/*dissonance*. If the scale of *consonance*/*dissonance* outlined in Atanassov et al. [21] might be considered as a scale of “quarters”, here a kind of scale of “thirds” is intentionally considered, corresponding to *α* = 0.67 (approximately two-thirds) and *β* = 0.33 (approximately one-third). For better understanding, Figure 1 represents the respective interpretative intuitionistic triangle.

In this case, the pair of criteria are said to be in one of the following:

- *positive consonance*, whenever $\mu_{C_{k},C_{l}}$ ≥ 2/3 and $\nu_{C_{k},C_{l}}$ < 1/3;
- *negative consonance*, whenever $\mu_{C_{k},C_{l}}$ < 1/3 and $\nu_{C_{k},C_{l}}$ ≥ 2/3;
- *dissonance*, whenever 0 ≤ $\mu_{C_{k},C_{l}}$ < 2/3, 0 ≤ $\nu_{C_{k},C_{l}}$ < 2/3, and 2/3 ≤ $\mu_{C_{k},C_{l}}$ + $\nu_{C_{k},C_{l}}$ ≤ 1;

and

- *uncertainty*, 0 ≤ $\mu_{C_{k},C_{l}}$ < 2/3, 0 ≤ $\nu_{C_{k},C_{l}}$ < 2/3, and 0 ≤ $\mu_{C_{k},C_{l}}$ + $\nu_{C_{k},C_{l}}$ < 2/3.

As seen in Figure 1, the presented scale of “thirds” preserves the symmetry in the interpretative intuitionistic triangle in accordance with the original “quarters”-based symmetry [21]. Thus, the combination *α* = 0.67 and *β* = 0.33 appears to be an appropriate selection in this investigation. It should be noted that such a systematic investigation of the impact of different threshold values for the *consonance* and *dissonance* intervals in the ICrA is conducted for the first time in the current study and may be considered as a methodological contribution to the presented analysis.

According to the pairwise performance comparison of the MOE scoring functions (Table 3), the following results can be outlined:

(1) an absence of any kind of agreement in all explored values of *α* and *β* with the experimental data (except one, between GBVI/WSA dG and (−log*K_d_*) or (−log*K_i_*) at *α* = 0.60 and *β* = 0.40, rows highlighted in grey in Table 3). This result is in accordance with our previous studies [19]. The lack of any agreement might also be explained by the fact that, even when implementing a variety of scoring terms and becoming more sophisticated, the scoring functions are still a computational approximation mostly aimed at assisting in the prediction of ligand binding poses. This is confirmed by the results of the BestRMSD docking output (Table 2).
(2) a *positive consonance* between two scoring functions, Alpha HB and London dG: in particular, for 0.67/0.33 threshold values, they are comparable in all four docking outputs (a row in bold in Table 3). The result suggests that these scoring functions might be used interchangeably. At the same time, some pairs show small comparability (Affinity dG–London dG and GBVI/WSA dG–London dG), suggesting that they can complement each other in consensus docking studies.

**Figure 2:**
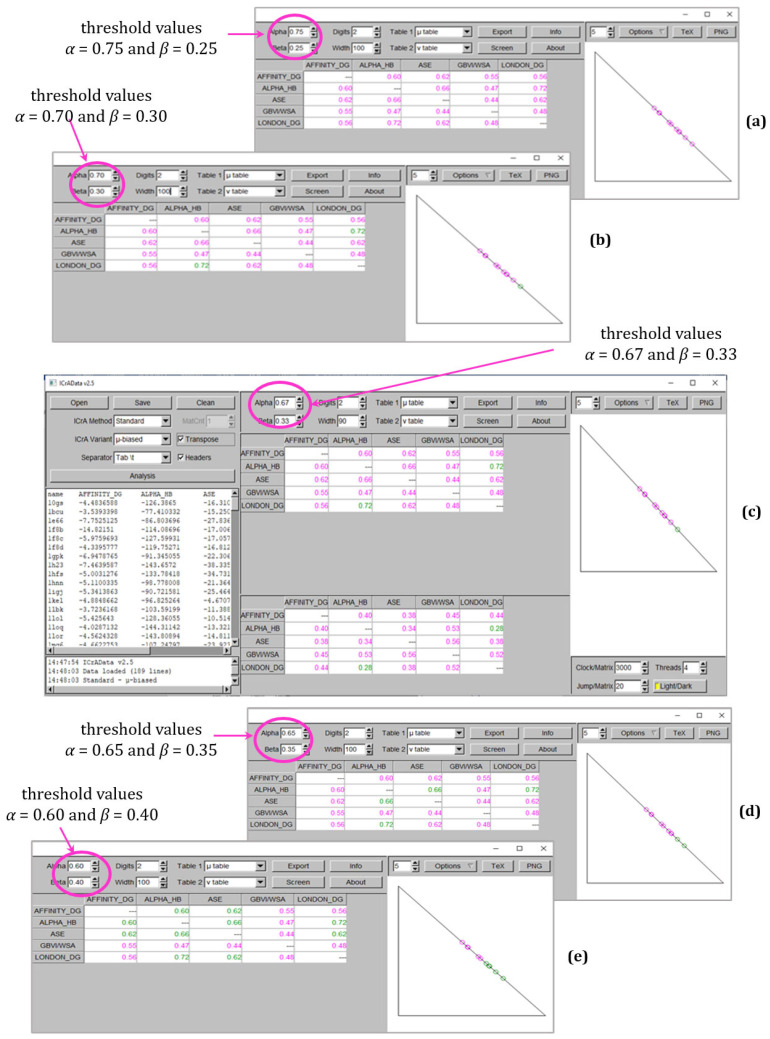
ICrA implementation with different values of *α* and *β* thresholds to assess the scoring functions based on BestDS as follows: (**a**) *α* = 0.75 and *β* = 0.25; (**b**) *α* = 0.70 and *β* = 0.30; (**c**) *α* = 0.67 and *β* = 0.33, (**d**) *α* = 0.65 and *β* = 0.35; and (**e**) *α* = 0.60 and *β* = 0.40.

**Figure 3:**
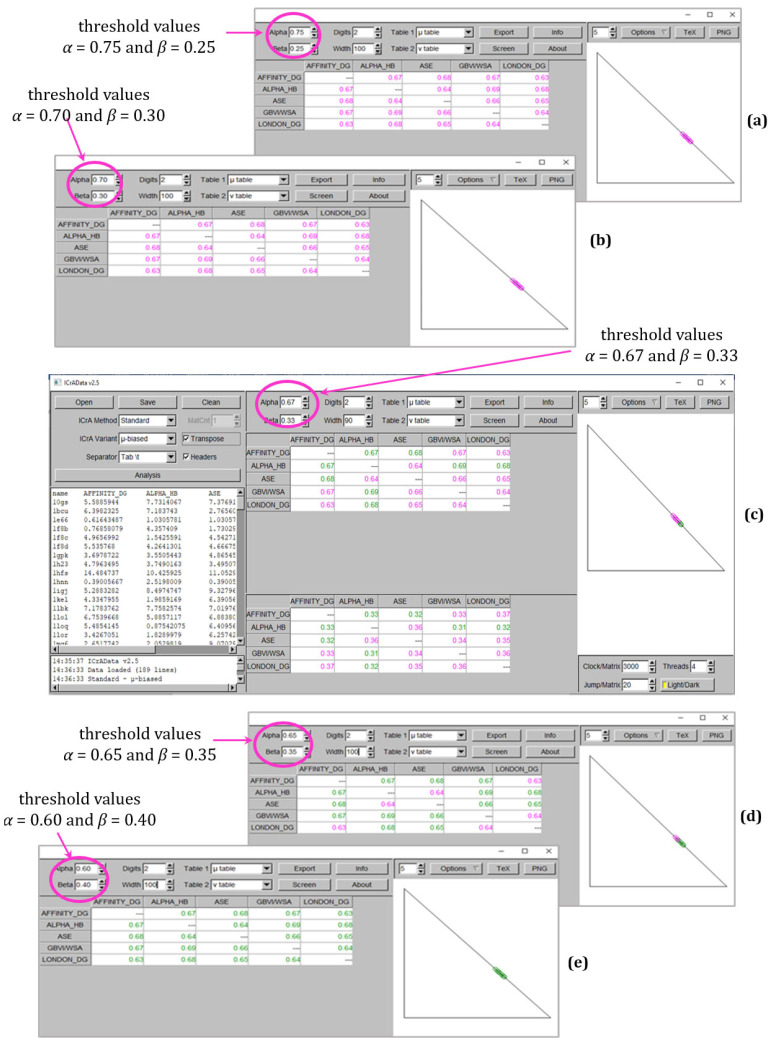
ICrA implementation with different values of *α* and *β* thresholds to assess the five scoring functions based on RMSD_BestDS as follows: (**a**) *α* = 0.75 and *β* = 0.25; (**b**) *α* = 0.70 and *β* = 0.30; (**c**) *α* = 0.67 and *β* = 0.33, (**d**) *α* = 0.65 and *β* = 0.35; and (**e**) *α* = 0.60 and *β* = 0.40.

Figure 2 and Figure 3 demonstrate the results from ICrA implementation for the explored values of thresholds *α* and *β*, respectively, for BestDS and RMSD_BestDS. Both figures show the ICrAData screenshots at conditionally defined threshold values of *α* = 0.75 and *β* = 0.25 (subplot a), *α* = 0.70 and *β* = 0.30 (subplot b), *α* = 0.67 and *β* = 0.33 (subplot c), *α* = 0.65 and *β* = 0.35 (subplot d), and *α* = 0.60 and *β* = 0.40 (subplot e). Subplot (c) represents the full screenshot from the ICrAData software, while the other subplots represent only the results for the degrees of agreement *µ* and the intuitionistic fuzzy triangle at different values of thresholds.

As seen in Figure 2, applying new values of thresholds leads to the appearance of pairs in *positive consonance*. For *α* = 0.70 and *β* = 0.30 (Figure 2b) and *α* = 0.67 and *β* = 0.33 (Figure 2c), *positive consonance* appears between the scoring functions Alpha HB and London dG, with no identified significant relations with the conditionally defined threshold values (Figure 2a). Further decreasing the difference between *α* and *β* leads to one more pair in *positive consonance*—Alpha HB–ASE for *α* = 0.65 and *β* = 0.35 (Figure 2d), and additionally three more pairs in *positive consonance*—namely Affinity dG–Alpha HB, Affinity dG–ASE, and ASE–London dG at *α* = 0.60 and *β* = 0.40 (Figure 2e).

As seen in Figure 3 and Table 1, RMSD_BestDS has the highest number of significant relations that appear when the new values of the thresholds are applied. Altogether, five pairs of scoring functions show *positive consonance* at *α* = 0.67 and *β* = 0.33, namely, Affinity dG–Alpha HB, Affinity dG–ASE, Affinity dG–GBVI/WSA dG, Alpha HB–GBVI/WSA dG, and Alpha HB–London dG (Figure 3c), in comparison to no significant relations identified at the conditionally defined threshold values (Figure 3a) of *α* = 0.70 and *β* = 0.30 (Figure 3b). Further decreasing the difference between α and β leads to two more pairs in *positive consonance*—between ASE–GBVI/WSA dG and ASE–London dG at *α* = 0.65 and *β* = 0.35 (Figure 3d), and even to three additional pairs between Affinity dG–London dG, Alpha HB–ASE, and GBVI/WSA dG–London dG at *α* = 0.60 and *β* = 0.40 (Figure 3e).

As mentioned above, the most “varicolored” picture from ICrA implementation is when the BestRMSD is considered (the results are presented in Supplementary Tables S1–S5). As seen in Table 1, *positive consonance* was observed for almost all pairs of scoring functions, while the other two pairs of scoring functions, ASE–GBVI/WSA dG and GBVI/WSA dG–London dG, are very close to the range of *positive consonance*. Then, still at the threshold values *α* = 0.70 and *β* = 0.30, all pairs of scoring functions show *positive consonance*. Based on this analysis, one may conclude that, according to the BestRMSD, all scoring functions give quite similar results.

For the DS_BestRMSD output, only one pair of scoring functions, Alpha HB–London dG, falls into the interval of *positive consonance* (Table 1 and Table 2; whole information is available in Supplementary Tables S1–S5,) when applying values of thresholds *α* = 0.70 and *β* = 0.30. Further decreases in the difference between *α* and *β* lead to three more pairs in *positive consonance*—between Affinity dG–GBVI/WSA dG, Alpha HB–ASE, and ASE–London dG, only at *α* = 0.60 and *β* = 0.40.

*Positive consonance* between the scoring functions Alpha HB and London dG was outlined in all investigated docking outputs. The close comparability between London dG and Alpha HB could be related to the fact that both functions include explicit terms related to hydrogen bonding. This type of protein–ligand interaction is among the most common, and it typically has a significant contribution to the binding energy of the protein–ligand complex. In addition, both scoring functions include terms that implicitly account for the conformational changes in the ligands upon binding to achieve the best fit of the ligand in the protein active site. In the case of London dG, this is estimated by the loss of ligand flexibility (calculated from ligand topology only); for Alpha HB, the geometrical fit to the protein active site is estimated by summing up the attractive and repulsive interactions between the protein and ligand atoms. In addition, London dG accounts for metal ligation and certain entropic effects (the average gain/loss of rotational and translational entropy and desolvation effects). The decision on which scoring function to use could be made based on the preliminary analysis of the protein–ligand interactions in the experimental complex of interest.

**Figure 4:**
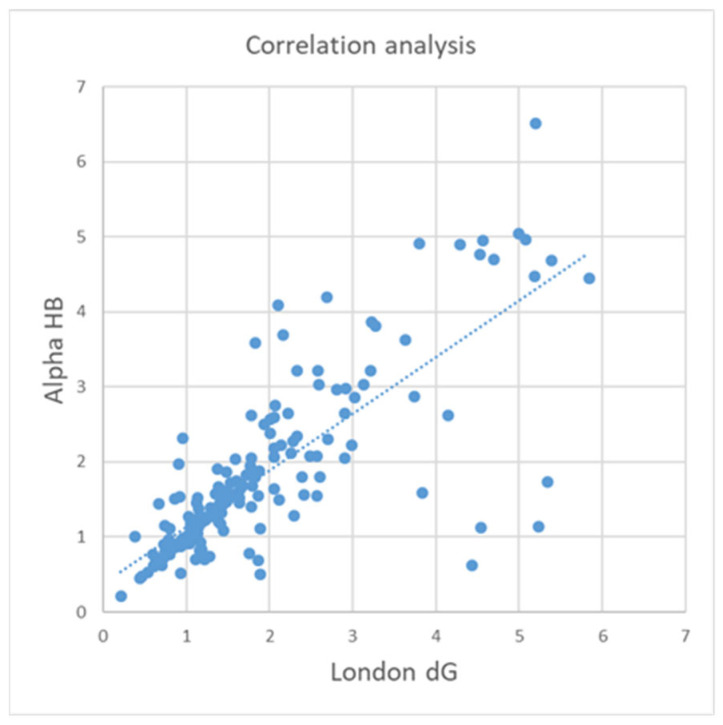
Correlation plot for Alpha HB and London dG based on the BestRMSD.

For the completeness of the comparison of the five scoring functions, a correlation analysis (CA) has also been performed. Table 4 summarizes the results obtained by the ICrA (the degrees of agreement *µ* are reported) and CA (Pearson correlation coefficient *R*) for all docking outputs. The CA shows a higher correlation only for BestRMSD, while for the other docking outputs, the observed correlations are relatively low. In the case of BestRMSD, the highest correlation coefficients coincide with the highest values of ICrA degrees of agreement. In particular, the relation between Alpha HB and London dG is evaluated with the highest values of degree of agreement by ICrA and with the second-best correlation coefficient by CA. Figure 4 illustrates the correlation in terms of CA between Alpha HB and London dG for BestRMSD (Table 4, correlation coefficient 0.79).

The absence of correlation (estimated by the Pearson correlation coefficients) between the docking scores and the experimental data on the binding affinity of the ligands in the complexes is not surprising. This result could not be related only to the heterogeneity of the experimental data (different measures of affinity, different methods and experimental protocols, various protein–ligand complexes) in the dataset used but rather to the fact that molecular docking has not initially been designed for the correlation of docking scores with experimental binding affinities [22]. The absence of such correlations has also been confirmed in our previous studies employing more consistent experimental data on the binding affinities of a homologous series of 88 benzamidine-type ligands toward thrombin, trypsin, and factor Xa [19]. Similar findings have been reported in systematic evaluations of scoring functions by other research groups. Notably, Li et al. [12] assessed a panel of 20 scoring functions, including those implemented in MOE, using the same CASF-2013 dataset. They concluded that the investigated scoring functions generally performed better at predicting binding poses than affinity assessments. These results are in accordance with our findings on the best-performing docking output (BestRMSD) and the absence of consonance with the experimental values; however, in contrast to [12], we confirmed these results by estimating similarity/dissimilarity between the scoring functions. In this way, our analysis adds a new value to the comparison of the scoring functions.

As seen in Table 4 and Figure 4, ICrA may offer enhanced capabilities over CA by enabling the identification of additional relationships among the evaluated scoring functions. ICrA, as well as CA, reports the degree of coincidence (in terms of ICrA, this is *positive consonance*), and both CA and ICrA report negative correlation (*negative consonance* in terms of ICrA), in which the values for one criterion increase while at the same time the values of the other criterion decrease. Unlike CA, ICrA also allows for classifying the criteria relations in *dissonance*, as well as accounting for the degree of uncertainty, which represents the advantage of ICrA over CA.

In this way, the results from ICrA implementation for the pairwise performance comparison of docking scoring functions would allow the user to decide on the selection of an appropriate scoring function in order to optimize the computational costs. This is relevant, especially when applied to virtual screening tasks. If two or more scoring functions produce similar results, only one of them can be used; instead, the scoring functions that give different results can be combined in consensus docking studies.

## 3. Materials and Methods

### 3.1. Dataset

**Figure 5:**
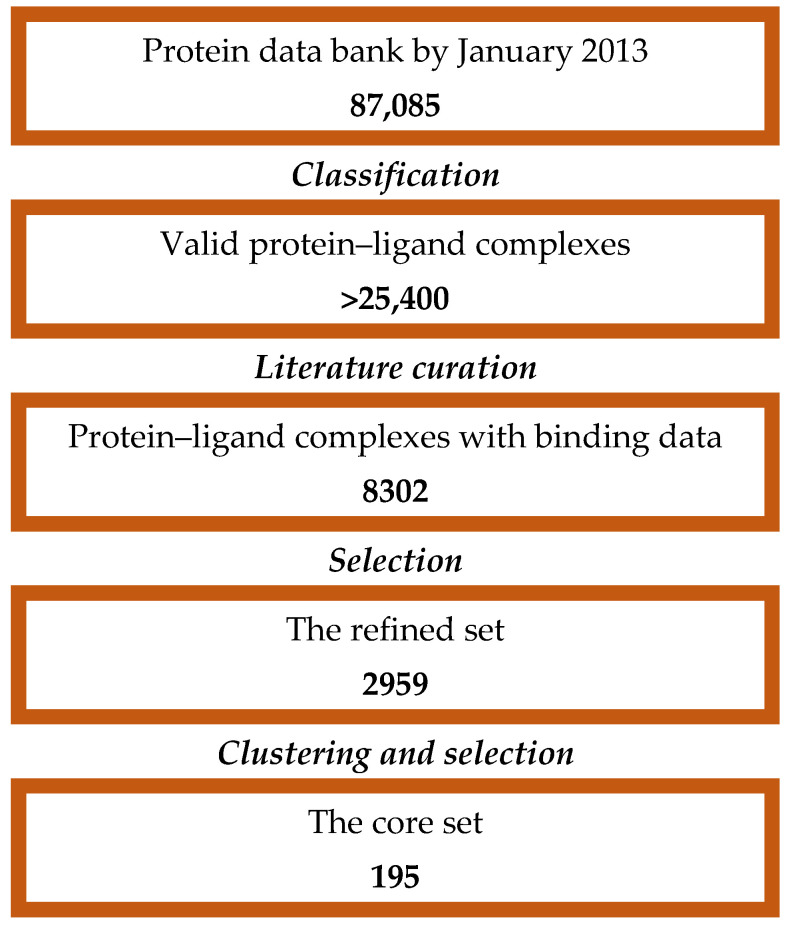
CASF-2013 dataset selection (adapted from [3]).

In the current investigation, CASF-2013 was selected as a benchmark dataset widely recognized by researchers dealing with the development and assessment of scoring functions in structure-based studies due to its representativeness, diversity, and quality of complex structures and binding affinity data [3] (Figure 5).

The final CASF-2013 dataset consists of 195 protein–ligand complexes with binding affinity data selected out of 8302 protein–ligand complexes recorded in the PDBbind database (https://www.pdbbind-plus.org.cn/casf, v. 2013, accessed on 22 May 2025). The qualified complexes were classified in 65 clusters by 90% similarity in protein sequences. Three representative complexes are chosen from each cluster to control sample redundancy.

### 3.2. Molecular Docking

MOE v. 2022 was used for the molecular docking studies. Prior to re-docking, all 195 protein–ligand complexes were protonated using the Protonate3D tool in MOE. The tool assigns hydrogens to structures following the proton geometry with optimal free energy and the ionization states of titratable protein groups. The rigid protein/flexible ligand option was used. The active site was defined by ligand atoms, and the water molecules were removed from the binding sites of the studied proteins. The placement was obtained by a triangle matcher algorithm.

Re-docking of the ligands in the protein–ligand complexes was performed, applying all available scoring functions in MOE v. 2022, namely London dG, ASE, Affinity dG, Alpha HB, and GBVI/WSA dG, as briefly described below [8,9]:

- ASE is based on the Gaussian approximation and depends on the radii of the atoms and the distance between the ligand atom and receptor atom pairs. ASE is proportional to the sum of the Gaussians over all ligand atom–receptor atom pairs.
- Affinity dG is a linear function that calculates the enthalpy contribution to the binding free energy, including terms based on interactions between H-bond donor and acceptor pairs, ionic interactions, metal ligation, hydrophobic interactions, unfavorable interactions (between hydrophobic and polar atoms,) and favorable interactions (between any two atoms).
- Alpha HB is a linear combination of two terms: (i) the geometric fit of the ligand to the binding site with regard to the attraction and repulsion depending on the distance between the atoms and (ii) H-bonding effects.
- London dG estimates the free binding energy of the ligand, accounting for the average gain or loss of rotational and translational entropy, the loss of flexibility of the ligand, the geometric imperfections of H-bonds and metal ligations compared to the ideal ones, and the desolvation energy of atoms.
- GBVI/WSA dG estimates the free energy of ligand bindings considering the weighted terms for the Coulomb energy, solvation energy, and van der Waals contributions.

Up to 30 poses per ligand were saved for each of the protein–ligand complexes and each of the investigated scoring functions.

### 3.3. InterCriteria Analysis Approach

The ICrA approach developed by Atanassov et al. in 2014 [16] is based on two mathematical formalisms: index matrices (IMs) [23] and intuitionistic fuzzy sets (IFSs) [24]. The algebraic apparatus of IMs allows for the processing of data arrays of diverse dimensions, while the IFS is a mathematical tool for handling uncertainty. By relying on IMs and IFSs, the ICrA allows for the identification of intercriteria relations in terms of *consonance* or *dissonance* between each pair of criteria, thus differentiating from the classical correlation analysis.

In the concept of IFSs [17], Atanassov builds the ICrA on Zadeh’s theory of fuzzy sets [25], which, on their side, are an extension of the classical notion of set. In classical set theory, an element either belongs or does not belong to the set; therefore, the membership of the element to the set is represented by the values 0 for non-membership or 1 otherwise. The fuzzy set theory introduces a degree of membership *μ* of the element *x* to the set, such that *μ* ∈ [0; 1]. The theory of IFSs further expands this notion by including the degree of non-membership *ν* of the element *x* to the set, such that *ν* ∈ [0; 1].

In mathematical terms, set *A* is defined as an intuitionistic fuzzy set as follows:

*A* = {⟨x, *μ*_A_(x), *ν*_A_(x)⟩|*x* ∈ *X*},

where *X* is the universum, and the two mappings *μ*_A_(x), *ν*_A_(x): *A* → [0, 1] are, respectively, the degree of membership and the degree of non-membership of each element *x* ∈ *X*, such that 0 ≤ *μ*_A_(x) + *ν*_A_(x) ≤ 1. The boundary conditions are 0 and 1, both giving the classical set.

The ICrA approach uses a two-dimensional (2D) IM as the input. The 2D IM is represented by a set indexing the rows, a set indexing the columns, and a set of elements corresponding to each pair of row and column index. In the case of ICrA, the IM can be written as [*O*, *C*, $e_{o,c}$], where *O* and *C* are, respectively, the set of row and column indices, *O* stands for objects, *C* stands for criteria, and $e_{o,c}$ corresponds to the evaluation of each object *O* against the criterion *C*. If we denote m as the number of objects and n as the number of criteria, the input IM for the ICrA can be represented as follows:

*C*_1_…*C_k_*…*C_n_**O*_1_$e_{O_{1},C_{1}}$…$e_{O_{1},C_{k}}$…$e_{O_{1},C_{n}}$………………*O_i_*$e_{O_{i},C_{1}}$…$e_{O_{i},C_{k}}$…$e_{O_{i},C_{n}}$………………*O_m_*$e_{O_{m},C_{1}}$…$e_{O_{m},C_{k}}$…$e_{O_{m},C_{n}}$

**Figure 6:**
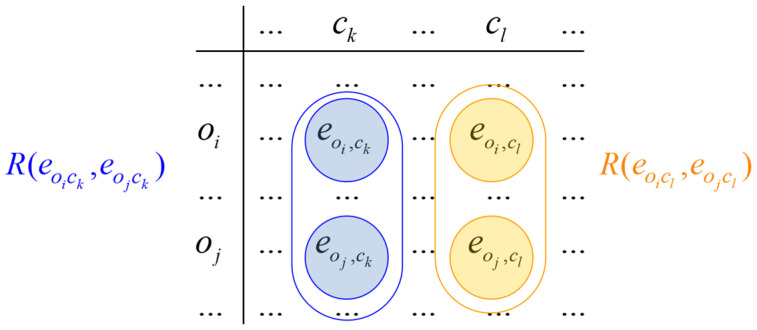
Formation of relations in the ICrA.

In the next step of the ICrA, relations are formed between every two elements of the matrix; thus, the evaluations of the criteria are compared in pairs for all objects in the matrix (Figure 6).

The relation $R(e_{O_{i},C_{k}},e_{O_{j},C_{k}})$ has dual relation $\bar{R}$, which is true in the cases when relation *R* is false and vice versa [16]. Two intuitionistic fuzzy counters, $S_{k,l}^{\mu }$ and $S_{k,l}^{\nu }$, are formed and are incremented based on the following rules:

– $S_{k,l}^{\mu }$ is the number of cases in which the relations $R(e_{O_{i},C_{k}},e_{O_{j},C_{k}})$ and $R(e_{O_{i},C_{l}},e_{O_{j},C_{l}})$ (or the relations $\bar{R}(e_{O_{i},C_{k}},e_{O_{j},C_{k}})$ and $\bar{R}(e_{O_{i},C_{l}},e_{O_{j},C_{l}})$) are simultaneously satisfied.
– $S_{k,l}^{\nu }$ is the number of cases in which the relation $R(e_{O_{i},C_{k}},e_{O_{j},C_{k}})$ and $\bar{R}(e_{O_{i},C_{l}},e_{O_{j},C_{l}})$ (or the relations $\bar{R}(e_{O_{i},C_{k}},e_{O_{j},C_{k}})$ and $R(e_{O_{i},C_{l}},e_{O_{j},C_{l}})$) are simultaneously satisfied.

The total number of pairwise comparisons between the evaluations of *m* objects is *m*(*m* − 1)/2; therefore, $0\leq S_{k,l}^{\mu }+S_{k,l}^{\nu }\leq \frac{m(m-1)}{2}$.

For every *k*, *l* (1 ≤ *k* ≤ *l* ≤ *m* and *m* ≥ 2), two normalized values are obtained from the above counters:

- $\mu_{C_{k},C_{l}}=2\frac{S_{k,l}^{\mu }}{m(m-1)}$, called the *degree of agreement* in terms of ICrA, and
- $\nu_{C_{k},C_{l}}=2\frac{S_{k,l}^{\nu }}{m(m-1)}$, called the *degree of disagreement* in terms of ICrA.

The pair $\langle \mu_{C_{k},C_{l}},\nu_{C_{k},C_{l}}\rangle$ constructed from these two numbers plays the role of intuitionistic fuzzy evaluation of the relation between any two criteria, *C_k_* and *C_l_*. The output IM puts together all these intuitionistic fuzzy pairs as follows:

*C*_1_…*C_k_*…*C_n_**C*_1_⟨1, 0⟩…$\langle \mu_{C_{1},C_{k}},\nu_{C_{1},C_{k}}\rangle$…$\langle \mu_{C_{1},C_{n}},\nu_{C_{1},C_{n}}\rangle$………………*C_k_*$\langle \mu_{C_{k},C_{1}},\nu_{C_{k},C_{1}}\rangle$…⟨1, 0⟩…$\langle \mu_{C_{k},C_{n}},\nu_{C_{k},C_{n}}\rangle$………………*C_n_*$\langle \mu_{C_{n},C_{1}},\nu_{C_{n},C_{1}}\rangle$…$\langle \mu_{C_{n},C_{k}},\nu_{C_{n},C_{k}}\rangle$…⟨1, 0⟩

The thresholds for $\mu_{C_{k},C_{l}}$ and $\nu_{C_{k},C_{l}}$ are determined algorithmically or are indicated by the user. Let *α*, *β* ∈ [0, 1] (with *α > β*) be the threshold values; then the pair of criteria *C_k_* and *C_l_* are said to be in one of the following:

- *positive consonance*, whenever $\mu_{C_{k},C_{l}}$ > *α* and $\nu_{C_{k},C_{l}}$ < *β*;
- *negative consonance*, whenever $\mu_{C_{k},C_{l}}$< *β* and $\nu_{C_{k},C_{l}}$ > *α*;
- *dissonance*, otherwise.

Further clarifications on determining *consonance* or *dissonance* can be found in [21].

### 3.4. Software Implementation of ICrA

A software application that implements the ICrA algorithm called ICrAData, v.2.5, was used during the calculations. It is freely available at https://intercriteria.net/software/ (accessed on 22 May 2025), along with the source code, a README file, and proper examples. The ICrAData interface allows for different variants of the ICrA and different algorithms for intercriteria relations calculations to be chosen, the input matrix to be read from a file or pasted from the clipboard, the input matrix to be transposed according to the investigation aims, the index matrices for the degrees of the agreements and disagreements to be visualized according to the user’s needs, the threshold values for *α* and *β* to be defined by the user (conditionally defined in ICrAData as *α* = 0.75 and *β* = 0.25, as introduced in [21]), the graphical interpretation of the results to be visualized by the intuitionistic fuzzy triangle, etc. For better visualization, the tables presenting index matrices for the degrees of the agreements and disagreements use colors for the cells’ values: green to indicate *positive consonance*, red for *negative consonance*, and magenta for the cases of *dissonance*.

### 3.5. Correlation Analysis

The correlations between every two variables investigated in this study were calculated in MS Excel using Pearson’s correlation coefficient (Pearson product moment correlation coefficient) [26].

## 4. Conclusions

Altogether, five scoring functions available in the MOE software package (London dG, Affinity dG, Alpha HB, ASE, and GBVI/WSA dG) were compared for their performance by using four docking outputs (BestDS, BestRMSD, RMSD_BestDS, and DS_BestRMSD) on the benchmark subset CASF-2013 consisting of 195 protein–ligand complexes from the PDBbind database.

The collected information was subjected to the ICrA using the ICrAData software and, additionally, to the CA. The most important outcomes from this investigation might be summarized as follows: (i) to the best of our knowledge, this is the first systematic investigation of the ICrA to assess the pairwise performance comparison of scoring functions available in a molecular software package, considering in parallel a variety of data outputs from molecular docking simulations; (ii) the analysis is also the first systematic investigation of the impact of different threshold values for the intervals of *consonance* and *dissonance* in terms of the ICrA; (iii) the scoring functions Alpha HB and London dG were outlined as a pair appearing in *positive consonance* in all investigated docking outputs, suggesting that these scoring functions might be used interchangeably; (iv) two pairs of scoring functions (Affinity dG–London dG and GBVI/WSA dG–London dG) were mostly in *dissonance* (except for BestRMSD), suggesting that they can complement each other in consensus docking studies; (v) for the docking output BestRMSD, all pairs of scoring functions were in *positive consonance,* thus confirming that the scoring functions studied perform best in reproducing the binding poses of the co-crystallized ligands; (vi) the comparison between the ICrA and CA results illustrates the ability of the ICrA compared to the CA to identify new relations between the investigated criteria.

The proposed approach can be applied to any other scoring functions and software packages as well as any other datasets of protein–ligand complexes of research interest.

## Figures and Tables

**Table 1:** Degrees of agreement *µ* obtained after ICrA application with the conditionally defined ICrAData threshold values of *α* = 0.75 and *β* = 0.25.

	BestDS	BestRMSD	RMSD_BestDS	DS_BestRMSD
**Affinity dG–Alpha HB**	0.60	0.81	0.67	0.59
**Affinity dG–ASE**	0.62	0.77	0.68	0.57
**Affinity dG–GBVI/WSA dG**	0.55	0.83	0.67	0.61
**Affinity dG–London dG**	0.56	0.78	0.63	0.56
**Alpha HB–ASE**	0.66	0.79	0.64	0.62
**Alpha HB–GBVI/WSA dG**	0.47	0.76	0.69	0.45
**Alpha HB–London dG**	0.72	0.84	0.68	0.70
**ASE–GBVI/WSA dG**	0.44	0.73	0.66	0.36
**ASE–London dG**	0.62	0.77	0.65	0.60
**GBVI/WSA dG–London dG**	0.48	0.73	0.64	0.46
**Affinity dG–(−log*K_d_*) or (−log*K_i_*)**	0.45	0.57	0.50	0.53
**Alpha HB–(−log*K_d_*) or (−log*K_i_*)**	0.40	0.53	0.44	0.49
**ASE–(−log*K_d_*) or (−log*K_i_*)**	0.35	0.56	0.37	0.57
**GBVI/WSA dG–(−log*K_d_*) or (−log*K_i_*)**	0.58	0.56	0.60	0.55
**London dG–(−log*K_d_*) or (−log*K_i_*)**	0.45	0.53	0.45	0.48

**Table 2:** Number of scoring function pairs in *positive consonance* for each docking output.

α/β	BestDS	BestRMSD	RMSD_BestDS	DS_BestRMSD
**0.75/0.25**	0	8	0	0
**0.70/0.30**	1	10	0	1
**0.67/0.33**	1	10	5	1
**0.65/0.35**	2	10	7	1
**0.60/0.40**	5	10	11	4

**Table 3:** Number of docking outputs in *positive consonance* for each pair of scoring functions.

Pairs of Scoring Functions	*α/* *β*				
	0.75/0.25	0.70/0.30	0.67/0.33	0.65/0.35	0.60/0.40
**Affinity dG–Alpha HB**	1	1	2	2	3
**Affinity dG** **–ASE**	1	1	2	2	3
**Affinity dG** **–GBVI/WSA dG**	1	1	2	2	3
**Affinity dG** **–London dG**	1	1	1	1	2
**Alpha HB** **–ASE**	1	1	1	2	4
**Alpha HB** **–GBVI/WSA dG**	1	1	2	2	2
**Alpha HB** **–London dG**	**1**	**3**	**4**	**4**	**4**
**ASE** **–GBVI/WSA dG**		1	1	2	2
**ASE** **–London dG**	1	1	1	2	4
**GBVI/WSA dG** **–London dG**		1	1	1	2
**Affinity dG** **–(−log*K_d_*) or (−log*K_i_*)**	0	0	0	0	0
**Alpha HB** **–(−log*K_d_*) or (−log*K_i_*)**	0	0	0	0	0
**ASE** **–(−log*K_d_*) or (−log*K_i_*)**	0	0	0	0	0
**GBVI/WSA dG** **–(−log*K_d_*) or (−log*K_i_*)**	0	0	0	0	1
**London dG** **–(−log*K_d_*) or (−log*K_i_*)**	0	0	0	0	0

**Table 4:** Results obtained after ICrA and CA applications.

	BestDS		BestRMSD		RMSD_BestDS		DS_BestRMSD	
	ICrA *µ*	CA *R*	ICrA *µ*	CA *R*	ICrA *µ*	CA *R*	ICrA *µ*	CA *R*
**Affinity dG** **–Alpha HB**	0.60	0.20	0.81	0.74	0.67	0.55	0.59	0.19
**Affinity dG** **–ASE**	0.62	0.23	0.77	0.68	0.68	0.53	0.57	0.03
**Affinity dG** **–GBVI/WSA dG**	0.55	0.12	0.83	0.81	0.67	0.55	0.61	0.22
**Affinity dG** **–London dG**	0.56	0.14	0.78	0.66	0.63	0.35	0.56	0.06
**Alpha HB** **–ASE**	0.66	0.54	0.79	0.77	0.64	0.45	0.62	0.42
**Alpha HB** **–GBVI/WSA dG**	0.47	−0.10	0.76	0.63	0.69	0.53	0.45	−0.05
**Alpha HB** **–London dG**	0.72	0.55	0.84	0.79	0.68	0.52	0.70	0.47
**ASE** **–GBVI/WSA dG**	0.44	−0.19	0.73	0.57	0.66	0.48	0.36	−0.16
**ASE** **–London dG**	0.62	0.29	0.77	0.68	0.65	0.42	0.60	0.24
**GBVI/WSA dG** **–London dG**	0.48	−0.16	0.73	0.57	0.64	0.36	0.46	−0.06
**Affinity dG–(−log*K_d_*) or (−log*K_i_*)**	0.45	−0.08	0.57	0.21	0.50	0.19	0.53	0.06
**Alpha HB** **–** **(−log*K_d_*) or (−log*K_i_*)**	0.40	−0.29	0.53	0.10	0.44	0.03	0.49	−0.18
**ASE** **–** **(−log*K_d_*) or (−log*K_i_*)**	0.35	−0.42	0.56	0.12	0.37	0.24	0.57	−0.37
**GBVI/WSA dG** **–** **(−log*K_d_*) or (−log*K_i_*)**	0.58	0.13	0.56	0.23	0.60	0.19	0.55	0.09
**London dG** **–** **(−log*K_d_*) or (−log*K_i_*)**	0.45	−0.13	0.53	0.06	0.45	−0.02	0.48	−0.11

## Data Availability

The original contributions presented in the study are included in the article and Supplementary Material. Further inquiries can be directed to the corresponding author.

## Supplementary Materials

The following supporting information can be downloaded at: https://www.mdpi.com/article/10.3390/molecules30132777/s1: Table S1: Results obtained after ICrA application at *α* = 0.75 and *β* = 0.25; Table S2: Results obtained after ICrA application at α = 0.70 and β = 0.30; Table S3: Results obtained after ICrA application at *α* = 0.67 and *β* = 0.33; Table S4: Results obtained after ICrA application at *α* = 0.65 and *β* = 0.35; Table S5: Results obtained after ICrA application at *α* = 0.60 and *β* = 0.40.
