# Supplementary material for: Pairwise Performance Comparison of Docking Scoring Functions: Computational Approach Using InterCriteria Analysis
Source: Molecules. 2025 Jun 27;30(13):2777. doi: 10.3390/molecules30132777 (PMC12250715; doi:10.3390/molecules30132777)
Supplement: Supplementary file 1 [file molecules-30-02777-s001.zip › molecules-3687295-supplementary.pdf]

Pairwise Performance Comparison of Docking Scoring Functions: Computational Approach Using InterCriteria Analysis

Maria Angelova, Petko Alov, Ivanka Tsakovska, Dessislava Jereva, Iglika Lessigiarska, Krassimir Atanassov, Ilza Pajeva and Tania Pencheva*
*Correspondence: *tania.pencheva@biomed.bas.bg*

Tables S1-S5 present the results obtained after ICrA application at different values of the thresholds α and β. Tables differ from each other only in revealed relations between the investigated scoring functions according to considered thresholds, but not in values, already calculated by ICrA. Colors in Tables S1-S5 reproduce the ones used in ICrAData, which are green for *positive consonance* (PC), red for *negative consonance*, and magenta for *dissonance*.

Table S1 Results obtained after ICrA application at α = 0.75 and β = 0.25

	BestDS	BestRMSD	RMSD_BestDS	DS_BestRMSD	Number of docking outputs in PC
**Affinity dG - Alpha HB**	0.60	0.81	0.67	0.59	1
**Affinity dG - ASE**	0.62	0.77	0.68	0.57	1
**Affinity dG - GBVI/WSA dG**	0.55	0.83	0.67	0.61	1
**Affinity dG - London dG**	0.56	0.78	0.63	0.56	1
**Alpha HB - ASE**	0.66	0.79	0.64	0.62	1
**Alpha HB - GBVI/WSA dG**	0.47	0.76	0.69	0.45	1
**Alpha HB - London dG**	0.72	0.84	0.68	0.70	1
**ASE - GBVI/WSA dG**	0.44	0.73	0.66	0.36	
**ASE - London dG**	0.62	0.77	0.65	0.60	1
**GBVI/WSA dG - London dG**	0.48	0.73	0.64	0.46	
**Affinity dG - (-logK_d) or (-logK_i)**	0.45	0.57	0.50	0.53	
**Alpha HB - (-logK_d) or (-logK_i)**	0.40	0.53	0.44	0.49	
**ASE - (-logK_d) or (-logK_i)**	0.35	0.56	0.37	0.57	
**GBVI/WSA dG - (-logK_d) or (-logK_i)**	0.58	0.56	0.60	0.55	
**London dG - (-logK_d) or (-logK_i)**	0.45	0.53	0.45	0.48	
**Number of pairs in PC**	0	8	0	0	

Table S2 Results obtained after ICrA application at $\alpha = 0.70$ and $\beta = 0.30$

	BestDS	BestRMSD	RMSD_BestDS	DS_BestRMSD	Number of docking outputs in PC
**Affinity dG - Alpha HB**	0.60	0.81	0.67	0.59	**1**
**Affinity dG - ASE**	0.62	0.77	0.68	0.57	**1**
**Affinity dG - GBVI/WSA dG**	0.55	0.83	0.67	0.61	**1**
**Affinity dG - London dG**	0.56	0.78	0.63	0.56	**1**
**Alpha HB - ASE**	0.66	0.79	0.64	0.62	**1**
**Alpha HB - GBVI/WSA dG**	0.47	0.76	0.69	0.45	**1**
**Alpha HB - London dG**	0.72	0.84	0.68	0.70	**3**
**ASE - GBVI/WSA dG**	0.44	0.73	0.66	0.36	**1**
**ASE - London dG**	0.62	0.77	0.65	0.60	**1**
**GBVI/WSA dG - London dG**	0.48	0.73	0.64	0.46	**1**
**Affinity dG - (-logK_d) or (-logK_i)**	0.45	0.57	0.50	0.53	
**Alpha HB - (-logK_d) or (-logK_i)**	0.40	0.53	0.44	0.49	
**ASE - (-logK_d) or (-logK_i)**	0.35	0.56	0.37	0.57	
**GBVI/WSA dG - (-logK_d) or (-logK_i)**	0.58	0.56	0.60	0.55	
**London dG - (-logK_d) or (-logK_i)**	0.45	0.53	0.45	0.48	
**Number of pairs in PC**	**1**	**10**	**0**	**1**	

Table S3 Results obtained after ICrA application at $\alpha = 0.67$ and $\beta = 0.33$

	BestDS	BestRMSD	RMSD_BestDS	DS_BestRMSD	Number of docking outputs in PC
**Affinity dG - Alpha HB**	0.60	0.81	0.67	0.59	**2**
**Affinity dG - ASE**	0.62	0.77	0.68	0.57	**2**
**Affinity dG - GBVI/WSA dG**	0.55	0.83	0.67	0.61	**2**
**Affinity dG - London dG**	0.56	0.78	0.63	0.56	**1**
**Alpha HB - ASE**	0.66	0.79	0.64	0.62	**1**
**Alpha HB - GBVI/WSA dG**	0.47	0.76	0.69	0.45	**2**
**Alpha HB - London dG**	0.72	0.84	0.68	0.70	**4**
**ASE - GBVI/WSA dG**	0.44	0.73	0.66	0.36	**1**
**ASE - London dG**	0.62	0.77	0.65	0.60	**1**
**GBVI/WSA dG - London dG**	0.48	0.73	0.64	0.46	**1**
**Affinity dG - (-logK_d) or (-logK_i)**	0.45	0.57	0.50	0.53	
**Alpha HB - (-logK_d) or (-logK_i)**	0.40	0.53	0.44	0.49	
**ASE - (-logK_d) or (-logK_i)**	0.35	0.56	0.37	0.57	
**GBVI/WSA dG - (-logK_d) or (-logK_i)**	0.58	0.56	0.60	0.55	
**London dG - (-logK_d) or (-logK_i)**	0.45	0.53	0.45	0.48	
**Number of pairs in PC**	**1**	**10**	**5**	**1**	

Table S4 Results obtained after ICrA application at $\alpha = 0.65$ and $\beta = 0.35$

	BestDS	BestRMSD	RMSD_BestDS	DS_BestRMSD	Number of docking outputs in PC
**Affinity dG - Alpha HB**	0.60	0.81	0.67	0.59	2
**Affinity dG - ASE**	0.62	0.77	0.68	0.57	2
**Affinity dG - GBVI/WSA dG**	0.55	0.83	0.67	0.61	2
**Affinity dG - London dG**	0.56	0.78	0.63	0.56	1
**Alpha HB - ASE**	0.66	0.79	0.64	0.62	2
**Alpha HB - GBVI/WSA dG**	0.47	0.76	0.69	0.45	2
**Alpha HB - London dG**	0.72	0.84	0.68	0.70	4
**ASE - GBVI/WSA dG**	0.44	0.73	0.66	0.36	2
**ASE - London dG**	0.62	0.77	0.65	0.60	2
**GBVI/WSA dG - London dG**	0.48	0.73	0.64	0.46	1
**Affinity dG - (-logK_d) or (-logK_i)**	0.45	0.57	0.50	0.53	
**Alpha HB - (-logK_d) or (-logK_i)**	0.40	0.53	0.44	0.49	
**ASE - (-logK_d) or (-logK_i)**	0.35	0.56	0.37	0.57	
**GBVI/WSA dG - (-logK_d) or (-logK_i)**	0.58	0.56	0.60	0.55	
**London dG - (-logK_d) or (-logK_i)**	0.45	0.53	0.45	0.48	
Number of pairs in PC	2	10	7	1	

Table S5 Results obtained after ICrA application at $\alpha = 0.60$ and $\beta = 0.40$

	BestDS	BestRMSD	RMSD_BestDS	DS_BestRMSD	Number of docking outputs in PC
**Affinity dG - Alpha HB**	0.60	0.81	0.67	0.59	3
**Affinity dG - ASE**	0.62	0.77	0.68	0.57	3
**Affinity dG - GBVI/WSA dG**	0.55	0.83	0.67	0.61	3
**Affinity dG - London dG**	0.56	0.78	0.63	0.56	2
**Alpha HB - ASE**	0.66	0.79	0.64	0.62	4
**Alpha HB - GBVI/WSA dG**	0.47	0.76	0.69	0.45	2
**Alpha HB - London dG**	0.72	0.84	0.68	0.70	4
**ASE - GBVI/WSA dG**	0.44	0.73	0.66	0.36	2
**ASE - London dG**	0.62	0.77	0.65	0.60	4
**GBVI/WSA dG - London dG**	0.48	0.73	0.64	0.46	2
**Affinity dG - (-logK_d) or (-logK_i)**	0.45	0.57	0.5	0.53	
**Alpha HB - (-logK_d) or (-logK_i)**	0.40	0.53	0.44	0.49	
**ASE - (-logK_d) or (-logK_i)**	0.35	0.56	0.37	0.57	
**GBVI/WSA dG - (-logK_d) or (-logK_i)**	0.58	0.56	0.60	0.55	
**London dG - (-logK_d) or (-logK_i)**	0.45	0.53	0.45	0.48	
Number of pairs in PC	5	10	11	4	
